# Clinical utility of the S3-score for molecular prediction of outcome in non-metastatic and metastatic clear cell renal cell carcinoma

**DOI:** 10.1186/s12916-018-1088-5

**Published:** 2018-07-05

**Authors:** Florian Büttner, Stefan Winter, Steffen Rausch, Jörg Hennenlotter, Stephan Kruck, Arnulf Stenzl, Marcus Scharpf, Falko Fend, Abbas Agaimy, Arndt Hartmann, Jens Bedke, Matthias Schwab, Elke Schaeffeler

**Affiliations:** 1 Dr. Margarete Fischer-Bosch Institute of Clinical Pharmacology, Auerbachstrasse 112, 70376 Stuttgart, Germany; 2 University of Tuebingen, Tuebingen, Germany; 3 Department of Urology, University Hospital Tuebingen, Tuebingen, Germany; 4 Institute of Pathology and Neuropathology, University Hospital Tuebingen, Tuebingen, Germany; 5 Institute of Pathology, Friedrich-Alexander University Erlangen-Nuernberg, University Hospital Erlangen-Nuernberg, Erlangen, Germany; 6 German Cancer Consortium (DKTK) and German Cancer Research Center (DKFZ), Heidelberg, Germany; 7 Department of Clinical Pharmacology, University Hospital Tuebingen, Tuebingen, Germany; 8 Department of Pharmacy and Biochemistry, University of Tuebingen, Tuebingen, Germany

**Keywords:** Renal cell carcinoma, Prognostic marker, Survival, ccRCC, Metastases, Sunitinib

## Abstract

**Background:**

Stratification of cancer patients to identify those with worse prognosis is increasingly important. Through in silico analyses, we recently developed a gene expression-based prognostic score (S3-score) for clear cell renal cell carcinoma (ccRCC), using the cell type-specific expression of 97 genes within the human nephron. Herein, we verified the score using whole-transcriptome data of independent cohorts and extend its application for patients with metastatic disease receiving tyrosine kinase inhibitor treatment. Finally, we sought to improve the signature for clinical application using qRT-PCR.

**Methods:**

A 97 gene-based S3-score (S3_97_) was evaluated in a set of 52 primary non-metastatic and metastatic ccRCC patients as well as in 53 primary metastatic tumors of sunitinib-treated patients. Gene expression data of The Cancer Genome Atlas (*n* = 463) was used for platform transfer and development of a simplified qRT-PCR-based 15-gene S3-score (S3_15_). This S3_15_-score was validated in 108 metastatic and non-metastatic ccRCC patients and ccRCC-derived metastases including in part several regions from one metastasis. Univariate and multivariate Cox regression stratified by T, N, M, and G were performed with cancer-specific and progression-free survival as primary endpoints.

**Results:**

The S3_97_-score was significantly associated with cancer-specific survival (CSS) in 52 ccRCC patients (HR 2.9, 95% Cl 1.0–8.0, *P*_Log-rank_ = 3.3 × 10^–2^) as well as progression-free survival in sunitinib-treated patients (2.1, 1.1–4.2, *P*_Log-rank_ = 2.2 × 10^–2^). The qRT-PCR based S3_15_-score performed similarly to the S3_97_-score, and was significantly associated with CSS in our extended cohort of 108 patients (5.0, 2.1–11.7, *P*_Log-rank_ = 5.1 × 10^–5^) including metastatic (9.3, 1.8–50.0, *P*_Log-rank_ = 2.3 × 10^–3^) and non-metastatic patients (4.4, 1.2–16.3, *P*_Log-rank_ = 1.6 × 10^–2^), even in multivariate Cox regression, including clinicopathological parameters (7.3, 2.5–21.5, *P*_Wald_ = 3.3 × 10^–4^). Matched primary tumors and metastases revealed similar S3_15_-scores, thus allowing prediction of outcome from metastatic tissue. The molecular-based qRT-PCR S3_15_-score significantly improved prediction of CSS by the established clinicopathological-based SSIGN score (*P* = 1.6 × 10^–3^).

**Conclusion:**

The S3-score offers a new clinical avenue for ccRCC risk stratification in the non-metastatic, metastatic, and sunitinib-treated setting.

**Electronic supplementary material:**

The online version of this article (10.1186/s12916-018-1088-5) contains supplementary material, which is available to authorized users.

## Background

Clear cell renal cell carcinoma (ccRCC) is the most common subtype of renal cell carcinoma, with a currently increasing incidence [1–3]. Approximately 30% of patients develop metastases and, despite the implementation of targeted therapies, the 5 year survival rate of patients with metastatic disease remains below 20%. Thus, stratification of patients with ccRCC into different molecularly defined groups to identify patients at risk of worse outcome is increasingly important in the perspective of personalized medicine. With this in mind, several prognostic scores have been developed based on, for example, pathological features, gene expression, or DNA methylation status [4–6]. One of the most widely applied score established on clinicopathological data is the SSIGN (stage, size, grade, and necrosis) score [7, 8], whereas the ClearCode34 score, which predicts two ccRCC subtypes (ccA/ccB), has been suggested for prediction of survival using gene expression data [9, 10]. Moreover, Rini et al. [11] proposed a 16-gene score to predict recurrence in ccRCC patients. In general, prognostic signatures using RNA-seq data hold great promise for precision oncology, as previously demonstrated for lung adenocarcinoma [12]. We recently developed an in silico prediction score (named S3-score) for ccRCC, based on the gene expression of 97 signature genes and the similarity of gene expression between tumor cells and their proposed normal cell of origin in the nephron [13]. The S3-score outperforms several other scores [13], including the ClearCode34 model, and significantly improves the predictive value of the SSIGN score and the original ccA/ccB assignment based on clustering [14]. Moreover, compared with the ccA/ccB signature, the S3-score is slightly less dependent on the tumor section investigated [13] and, in consequence displays little intra-tumor heterogeneity. This is of importance because, in a recent study investigating the ccA/ccB signature [10], approximately one-quarter of metastatic tumors (two of nine patients) displayed intra-tumor heterogeneity and, in 43% of the cases, patient-matched primary and metastatic tumors displayed different molecular ccA/ccB subtypes. In this context, a recent multiregion sampling process using a protein-based prognostic model was described, enabling the study of the impact of intra-tumor heterogeneity on risk stratification of sunitinib-treated metastatic patients [15].

As our S3-score was evaluated only in silico using data from The Cancer Genome Atlas (TCGA), we now intended to verify the performance of the score using newly generated whole transcriptome data of an independent cohort of ccRCC patients, including metastases derived from ccRCC, to determine the concordance of the score prediction in primary tumors and ccRCC-derived metastases. Moreover, we evaluated whether the score predicts outcome in sunitinib-treated ccRCC patients. Finally, our objective was to improve the clinical applicability of the S3-score by reducing the number of genes necessary for calculation of the score and by using the more cost-effective real-time PCR technology.

## Methods

### Study cohorts

The study investigated different ccRCC cohorts listed in Table 1 and Additional file 1: Figure S1.

**Table 1:** Patient demographics and clinical characteristics of The Cancer Genome Atlas (TCGA) clear cell renal cell carcinoma (ccRCC) cohort (*n* = 463 with available RNA-Seq data), as well as our cohorts (*n* = 52 with available microarray data; *n* = 108 with available RT-PCR data) and a sunitinib-treated cohort published by Beuselinck et al. [17]^a^ (*n* = 53)

		ccRCC TCGA (*n* = 463)		ccRCC cohort 1 (*n* = 52)		Sunitinib treated cohort^a^ (*n* = 53)		Extended ccRCC cohort 2^b^ (*n* = 108)	
		*n*, value	%	*n*, value	%	*n*, value	%	*n*, value	%
Sex	Male	297	64.15%	35	67.3%	37	69.81%	63	58.33%
	Female	166	35.85%	17	32.69%	16	30.19%	45	41.67%
Age (year)	Median (range)	61 (26–90)		64 (37–90)		58 (44–80)		65 (34–90)	
T	T1	226	48.81%	17	32.69%	NA	NA	51	47.22%
	T2	59	12.74%	4	7.69%	NA	NA	10	9.26%
	T3	168	36.29%	31	59.62%	NA	NA	47	43.52%
	T4	10	2.16%	0	0.00%	NA	NA	0	0.00%
N	N0	215	46.44%	46	88.46%	NA	NA	97	89.81%
	N1	15	3.24%	4	7.69%	NA	NA	8	7.41%
	N2	0	0.00%	2	3.85%	NA	NA	3	2.78%
	NX	233	50.32%	0	0.00%	NA	NA	0	0.00%
M	M0	374	80.78%	41	78.85%	NA	NA	92	85.19%
	M1	76	16.41%	10	19.23%	NA	NA	15	13.89%
	MX	13	2.81%	1	1.92%	NA	NA	1	0.93%
G	G1	7	1.51%	9	17.31%	NA	NA	24	22.22%
	G2	200	43.20%	30	57.69%	NA	NA	60	55.56%
	G2–G3	0	0.00%	1	1.92%	NA	NA	2	1.85%
	G3	183	39.52%	11	21.15%	NA	NA	20	18.52%
	G4	72	15.55%	1	1.92%	NA	NA	1	0.93%
	GX	1	0.22%	0	0.00%	NA	NA	0	0.00%
	NA	0	0.00%	0	0.00%	NA	NA	1	0.93%
Necrosis	Present	218	47.08%	10	19.23%	NA	NA	17	15.74%
	Absent	245	52.92%	42	80.77%	NA	NA	91	84.26%
Follow-up time (years)	Median (range)	3.1 (0.0–10.0)		3.0 (0.0–10.0)		1.0 (0.1–4.9)		3.4 (0.0–11.1)	
Overall survival	Deceased	152	32.83%	17	32.69%	NA	NA	29	26.85%
	Alive	311	67.17%	35	67.31%	NA	NA	79	73.15%
Cancer-specific survival	Cancer-related death	104	22.46%	15	28.85%	NA	NA	21	19.44%
	Alive/non-cancer-related death	359	77.54%	37	71.15%	NA	NA	87	80.56%
Progression free survival under sunitinib therapy	Yes	–	–	–	–	14	26.4%	–	–
	No	–	–	–	–	39	73.6%	–	–

*T* primary tumor, *N* regional lymph node, *M* distant metastasis present at diagnosis, *G* grading, *NA* not available

^a^ccRCC cohort published by Beuselinck et al. [17]; descriptive data were not available

^b^This extended ccRCC cohort 2 includes the 52 patients from ccRCC cohort 1

First, our 97 gene-based S3-score (S3_97_), which was developed using publically available gene expression data of a ccRCC cohort from TCGA (*n* = 463) (Table 1) [16] was evaluated in a set of 52 primary ccRCC patients (ccRCC cohort 1). These 52 primary tumor samples were collected from non-metastatic and metastatic patients with ccRCC histology, treated at the Department of Urology, University Hospital Tuebingen, Germany. Patient characteristics are provided in Table 1. The use of the tissue was approved by the ethics committee of the University of Tuebingen and informed written consent was provided by each subject prior to surgical resection. Cancer-specific survival (CSS) was used as the endpoint in the survival analysis of these ccRCC patients.

In addition, publicly available gene expression data from an independent cohort of primary tumors obtained from sunitinib-treated ccRCC patients (*n* = 53, sunitinib-treated cohort) (Table 1) [17] were used in the analysis. This cohort consisted of ccRCC patients with synchronous or metachronous metastases, who received first-line sunitinib treatment (dosing schedule: 50 mg/day, 4 weeks on/2 weeks off; at least one 28-day cycle of sunitinib treatment completed; prior cytokine therapy allowed) [17]. Primary ccRCC tissue samples were collected from patients undergoing nephrectomy prior to sunitinib treatment [17]. Further details of these study patients are outlined in Beuselinck et al. [17]. Progression-free survival was used as the endpoint in the survival analysis of sunitinib-treated ccRCC patients.

Next, publicly available gene expression data of TCGA [16] from the cohort of ccRCC patients (*n* = 463) (Table 1) were used as a development cohort to define a modified S3-score, which requires a reduced number of genes for clinical application. This S3_15_-score was validated in an extended cohort of 108 metastatic and non-metastatic ccRCC patients treated at the Department of Urology, University Hospital Tuebingen, Germany (extended ccRCC cohort 2, *n* = 108) (Table 1). CSS was used as endpoint in the survival analysis. Kaplan–Meier curves of CSS for ccRCC cohorts 1 and 2, as well as for the TCGA cohort are shown in Additional file 1: Figure S2.

In addition, metastases samples (*n* = 22) derived from 15 patients treated at the Department of Urology, University Hospital Tuebingen, Germany, were collected, including matched primary tumor and metastases samples from five patients of our ccRCC cohorts 1 and 2 (Additional file 1: Table S1 and S5). In part, several regions from one metastasis were collected. Further details about metastases are given in Additional file 1: Table S1 and S5. Use of the tissue was approved by the ethics committee of the University of Tuebingen and informed written consent was provided by each subject prior to surgical resection.

Additional file 1: Figure S1 shows an overview about the workflow of data analyses including the different cohorts and technologies used in the present study.

### Gene expression analyses and S3-score calculation

Total RNA was isolated from fresh-frozen ccRCC and metastasis tissue using the mirVana™ miRNA Isolation Kit (Life Technologies) as previously described [18, 19]. Genome-wide transcriptome analyses were performed using Human Transcriptome Array HTA 2.0 (Affymetrix) according to the manufacturer’s protocol. Further processing of microarray data were performed as previously described [18] (Additional file 1: Supplementary methods). Gene expression data (generated using HuGene 1.0ST Affymetrix array) from 53 sunitinib-treated ccRCC patients were downloaded from ArrayExpress (E-MTAB-3267).

Quantitative real-time PCR (qRT-PCR) was performed using TaqMan technology on a BioMARK System (Fluidigm) as described previously [18, 19]. TaqMan gene expression assays for 97 genes of the S3-score, as well as five genes used for normalization were purchased from Life Technologies. Further details about calculation of the S3-score based on interprofile correlations and development of a S3-score calculation model for use of qRT-PCR data are provided in the Additional file 1: Supplementary methods.

### ClearCode34 and SSIGN calculation

The SSIGN score was calculated as denoted in Zigeuner et al. [8]. The ClearCode34 classifier, as introduced by Brooks et al. [9], was applied on the set of matched primary tumors and metastases of our present cohort for which genome-wide expression data measured by HTA 2.0 microarrays were available (Additional file 1: Supplementary methods).

### Statistical analyses

All statistical analyses were performed with R-3.3.3, including additional packages (Additional file 1: Supplementary methods) [20]. Survival analyses for endpoints CSS or progression-free survival were conducted by Kaplan–Meier curves and corresponding log-rank tests as well as uni- and multivariate Cox models. Comparisons of Cox models were performed by analysis of deviance. All statistical tests were two sided. Statistical significance was defined as *P* < 0.05 (Additional file 1: Supplementary methods).

## Results

### Evaluation of the S3-score in ccRCC primary tumors and primary tumors of patients treated with sunitinib

**Fig. 1:**
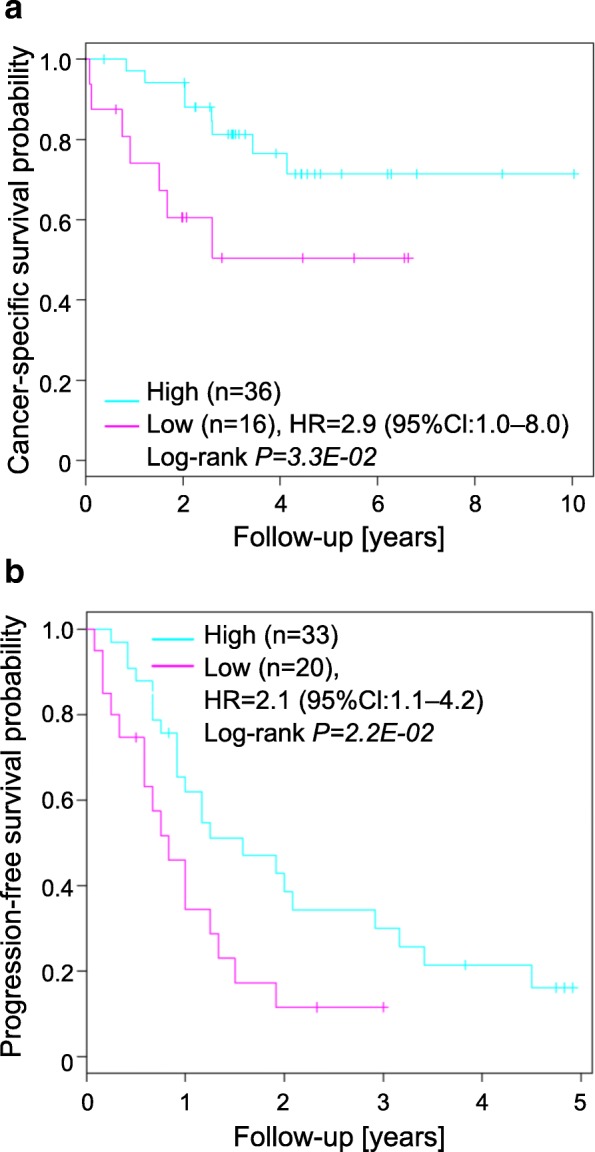
**a** Cancer-specific survival (CSS) of clear cell renal cell carcinoma (ccRCC) tumors predicted by the S3_97_-score. Kaplan–Meier curves showing CSS of ccRCC cohort 1 (*n* = 52). Groups are defined by the cut-off of the S3_97_-score, as determined by conditional inference tree models in Büttner et al. [13]. **b** Validation of the S3_97_-score in sunitinib-treated ccRCC patients. Kaplan–Meier curves showing progression-free survival predicted by the S3_97_-score in an independent cohort of primary tumors of sunitinib-treated patients (*n* = 53). Groups are defined by the same cut-off value of the S3_97_-score as in a. *HR* hazard ratio, *CI* confidence interval. Detailed information on statistical methods is provided in Additional file 1: Supplementary methods

We previously developed the S3-score in silico using RNA-seq data from the TCGA (Table 1) [13]. The S3-score was calculated based on 97 genes by correlating tumor expression to the expression in the eight nephron regions. In the present work, we first evaluated this 97 gene-based S3-score (S3_97_) in our own cohort, consisting of 52 ccRCC samples (ccRCC cohort1) (Table 1) for which genome-wide expression data using transcriptome arrays were generated. Partitioning of the ccRCC samples by means of the cut-off value that was established in our previous work [13] resulted in two groups with significantly varying CSS (Fig. 1a); i.e. patients with a high S3_97_-score had an decreased risk for cancer-related death compared to patients with low S3_97_-scores. Furthermore, univariate Cox regression including evaluation of the predictive ability according to Harrell’s c-index indicated a significant association of the S3_97_-score with patient survival (HR 2.9, 95% Cl 1.0–8.0, *P*_Log-rank_ = 3.3 × 10^–2^) (Additional file 1: Table S2).

Since survival prediction might be majorly influenced by treatment with, for example, sunitinib, we next investigated whether prediction of survival is possible in sunitinib-treated patients. Using a cohort of 53 sunitinib-treated metastatic ccRCC patients with publically available microarray data [17], we calculated the S3-score based on the 97 signature genes. Partitioning of the sunitinib-treated patients by means of our established cut-off value resulted in two groups with significantly varying progression-free survival (Fig. 1b); i.e., patients with a high S3_97_-score had increased progression-free survival probability after sunitinib treatment compared with patients with a low S3_97_-score. Furthermore, univariate Cox regression including evaluation of the predictive ability according to Harrell’s c-index, indicated a significant association of the S3_97_-score with patient survival after treatment with sunitinib (HR 2.1, 95% CI 1.1–4.2, *P*_Log−rank_ = 2.2 × 10^–2^) (Additional file 1: Table S2).

### Refinement of the 97 gene-based S3_97_-score for clinical application

Based on our results, the S3_97_-score has the ability to significantly predict not only CSS in ccRCC patients, but also the progression-free survival in sunitinib-treated individuals. However, calculation of the score was currently based on gene expression data of 97 marker genes, generated through genome-wide transcriptome analyses (RNA-seq or microarray). Thus, for clinical application and utility of the S3-score, expression analyses using quantitative real-time PCR (qRT-PCR), as well as a reduced number of genes, would be more appropriate. Therefore, we aimed to develop a new calculation model of the S3-score. First, the expression of the 97 signature genes, which constituted the basis for the development of the new prediction approach, and the expression of five normalization genes, was quantified by qRT-PCR in our extended ccRCC cohort of 108 non-metastatic and metastatic samples (ccRCC cohort 2) (Table 1). In order to ensure minimum failure rates in future applications, all assays that failed at least once were excluded. Moreover, we considered only genes that were (after normalization) comparable with respect to mean expression and variation of expression between the RNA-seq data from the TCGA cohort and the RT-PCR values (Additional file 1: Supplementary methods and Additional file 1: Figure S3). In total, the resulting set of variables used for model selection included 41 genes. Subsequently, a linear model using RNA-seq data from the TCGA cohort was created that reconstructs the correlation-based S3-scores.

**Fig. 2:**
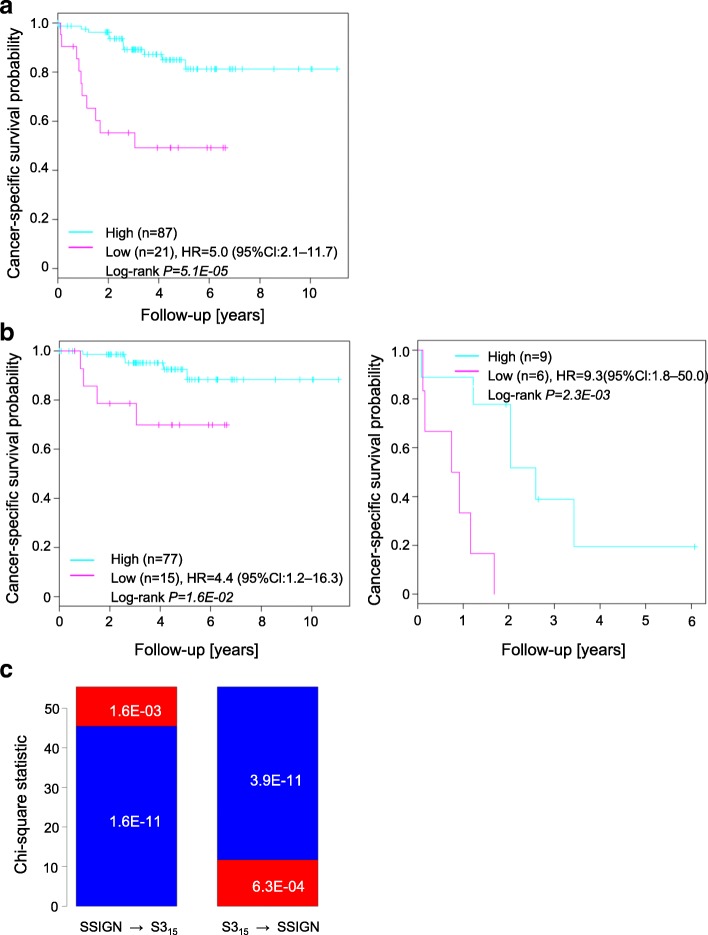
Cancer-specific survival (CSS) of clear cell renal cell carcinoma (ccRCC) tumors of our validation cohort predicted by the simplified RT-PCR-based S3_15_-score. Kaplan–Meier curves showing CSS of (**a**) the extended ccRCC cohort 2 (*n* = 108) and (**b**) the non-metastatic (*n* = 92) and metastatic subsets (*n* = 15) of this cohort. Groups are defined by the cut-off of the S3_15_-score, as determined by conditional inference tree models. **c** S3_15_-score significantly improves the established SSIGN prediction score. χ^2^ statistic values depict the improvement of the model likelihood when risk classification based on the S3_15_-score (red) was added to the Cox model initially including only the SSIGN score (blue; left) or vice versa (right). χ^2^ test *P* values are shown in the bars. *HR* hazard ratio, *CI* confidence interval. Detailed information on statistical methods is provided in Additional file 1: Supplementary methods

The resulting model identified by model selection included 15 genes (Additional file 1: Table S3) and showed good correlation with microarray-based values in our cohort (Spearman’s rank correlation coefficient = 0.91) (Additional file 1: Figure S4). Thus, including the five normalization genes, S3_15_-score determination based on qRT-PCR requires only 20 genes to be measured. Univariate Cox regression indicated that the S3_15_-score was significantly associated with CSS in our extended ccRCC cohort 2 (*n* = 108) (Table 2). CSS was significantly different between patients with a high and low S3_15_-score in the cohort (*n* = 108, HR 5.0, 95% Cl 2.1–11.7, *P*_Log-rank_ = 5.15 × 10^–5^) (Fig. 2a). Moreover, similarly to the 97 gene-based S3_97_-score we could confirm the ability of the S3_15_-score to predict CSS in non-metastatic (HR 4.4, 95% Cl 1.2–16.3, *P*_Log-Rank_ = 1.6 × 10^–2^) as well as metastatic patients (HR 9.3, 95% Cl 1.8–50.0, *P*_Log-rank_ = 2.3 × 10^–3^) (Fig. 2b).

**Table 2:** Univariate Cox regression for cancer-specific survival in the extended clear cell renal cell carcinoma cohort 2 (*n* = 108)

Univariate analyses	Variable	Level	No. of cases	HR (95% CI)	*P* value	c-index
					(Log-rank test)	
All patients	S3_15_-score	high	87	1 (Ref.)	5.15 × 10^–5^	0.69
		low	21	4.96 (2.10–11.72)		
M0	S3_15_-score	high	77	1 (Ref.)	1.62 × 10^–2^	0.68
		low	15	4.37 (1.17–16.29)		
M1	S3_15_-score	high	9	1 (Ref.)	2.31 × 10^–3^	0.71
		low	6	9.32 (1.75–49.58)		

*CI* confidence interval, *HR* hazard ratio, *Ref.* reference level

S3_15_-scores were determined based on gene expression data measured by RT-PCR; tumors with metastasis status MX were disregarded

As expected, a higher incidence of advanced stage tumors as well as metastatic tumors occurred in the S3_15_-low group with poor survival (Additional file 1: Figure S5 and Table S4). Next, we compared the S3_15_-score with clinicopathological prediction factors (T, N, M, G). Multivariate Cox regression indicated that the S3_15_-score is able to significantly improve the predictive ability of the clinicopathological parameters (Table 3). Additionally, the multivariate Cox model outperformed the univariate model (TNMG vs. TNMG+S3: *P*_χ2_ = 3.98 × 10^–4^). Moreover, the S3_15_-score significantly improved CSS prediction when added to the Cox model initially including only the clinicopathologic-based SSIGN score (*P*_χ2_ = 1.6 × 10^–3^) (Fig. 2c).

**Table 3:** Multivariate Cox regression for cancer-specific survival in the extended ccRCC cohort 2 (*n* = 108)

Multivariate analyses	variable	level	*P*-value	HR (95%CI)
			(Wald test)	
Including T,N,M,G and S3_15_-score	S3_15_-score	high		1 (Ref.)
		low	0.00033	7.3 (2.5–21.5)
	Primary tumor	T1		1 (Ref.)
		T2	0.37	3.0 (0.3–35.0)
		T3	0.01	6.0 (1.5–24.3)
	Lymph Nodes	N0		1 (Ref.)
		N1	0.25	0.5 (0.1–1.7)
		N2	0.45	0.4 (0.0–3.9)
	Distant metastasis	M0		1 (Ref.)
		M1	0.00001	23.5 (6.0–92.2)
	Fuhrman grade	G1		1 (Ref.)
		G2	0.73	1.5 (0.2–14.1)
		G3	0.93	0.9 (0.1–10.3)

*Abbreviations*: *CI* confidence interval, *HR* hazard ratio, *Ref.* reference level. S3_15_-scores were determined based on gene expression data measured by RT-PCR; T, primary tumor; N, regional lymph node; M, distant metastasis present at diagnosis; G, grading; tumors with grade “G2–3” and “G4” were added to “G3”. Tumors with no grading information or metastasis status “MX” were disregarded

### Evaluation of the S3_97_-score and S3_15_-score in metastases derived from ccRCC patients

**Fig. 3:**
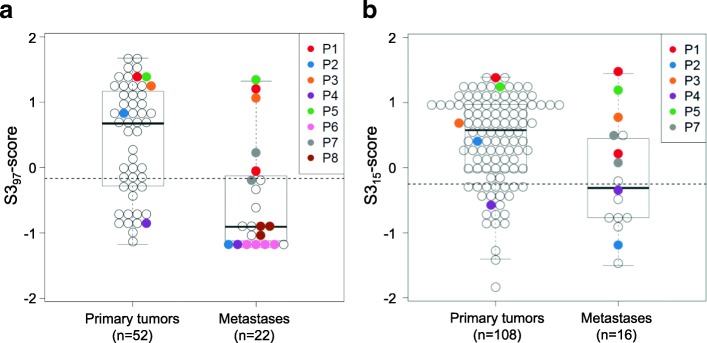
**a** S3_97_-score prediction in primary tumors and metastases samples derived from clear cell renal cell carcinoma (ccRCC) patients. Identical colors indicate primary and metastatic tissues derived from the same patient; for one patient (P6), four regions of one metastasis were analyzed. The dashed horizontal line indicates the S3_97_-score cut-off. **b** S3_15_-score in primary tumors and metastases, indicating similar scores in matched primary tumors and metastases except for one case. The dashed horizontal line indicates the S3_15_-score cut-off. Detailed information on statistical methods is provided in Additional file 1: Supplementary methods

Tumor heterogeneity of the original S3-score has been previously evaluated to assess whether a single tumor sample is sufficient for prediction of survival [13]. We now aimed to investigate the S3_97_-score and S3_15_-score in metastases in order to evaluate the concordance between primary tumors and metastases. First, we analyzed the S3_97_-score in metastases using microarray data. For a total of 15 ccRCC patients, genome-wide expression data were generated from metastases samples, including five metastatic patients from our ccRCC cohorts with matched primary tumor and metastases samples, as well as three patients for whom several metastases were available. Calculation of the S3_97_-score individually for tumor and metastases resulted in similar risk prediction (Fig. 3a). One patient (P4) was assigned to the high risk group (low S3_97_-score) with worse prognosis using either metastases or tumor tissue (Additional file 1: Table S5). Three patients (P1, P3, P5) showed a high S3_97_-score in tumor as well as metastasis tissue (Additional file 1: Table S5). S3-score was discordant between the primary tumor and its metastasis in only one sample (P2). For different metastases derived from the same patient (P7, P8), as well as for four regions of one metastasis (P6), the S3_97_-score values were also comparable (Fig. 3a). Using the ClearCode34 signature, recently used also for metastatic tissue [10], revealed similar results and classification of tumor/metastases pairs into different molecular subtypes as the S3_97_-score (Additional file 1: Table S5). We additionally performed qRT-PCR quantification and calculation of the S3_15_-score in a subset of metastases samples. Regarding the five metastatic patients from our ccRCC cohorts for whom primary tumors as well as metastases were available, we found that, except for one case, the S3_97_-score tendency was preserved using the improved S3_15_-score (Fig. 3b).

## Discussion

Several risk scores based on gene expression data have been developed for prediction of patient survival in ccRCC [4]. We recently developed a novel prediction score, named the S3-score, based on the similarity of gene expression in the tumor to its cell of origin in the nephron region [13, 21]. Thus, in contrast to other scores, risk prediction using the S3-score is related to biologic alterations of the cell of origin of ccRCC. The S3-score outperformed other scores or signatures based on gene expression data or clinicopathological variables [13, 21] and was even able to improve the predictive value of the clinically validated SSIGN score [7, 8]. Moreover, evaluation of tumor heterogeneity of our S3-score showed that only a few samples displayed heterogeneity [13], which indicates that risk prediction with our score is largely independent from the tumor region investigated.

Generally, most of the scores developed using gene expression data are thus far not introduced into clinical practice because they have not been generated to evaluate individual patients. Thus, for clinical application, the prediction scores need to be validated in several studies defining optimal cut-off values for classification of individual patients into subtypes. Moreover, prediction scores, typically developed using genome-wide gene expression data, need to be evaluated using different technologies and gene expression platforms. Since our S3-score, which is based on the expression of 97 signature genes, was originally developed using RNA-seq data from the TCGA, we first evaluated its predictive ability in the present work using gene expression data generated through microarray technology in our own ccRCC cohort. Here, we showed not only that a platform transfer to microarray data is possible, but also that the S3_97_-score significantly predicts CSS in our cohort.

In contrast to other prediction scores such as the 16-gene signatures [11], the 97 marker genes were not selected based on pathway analyses (e.g., including genes related to inflammation or immune response) and subsequent optimization for prediction of prognosis, but were originally selected to show that tumor aggressiveness in RCC correlates with the level of divergence from its cell of origin within the nephron region. Noticeably, we observe an overlap of one vascular pathway gene (PPAP2B) in the 97 marker genes and those genes from the 16-gene signature described by Rini et al. [11]. Further studies are warranted to compare the predictive ability of both scores.

Because metastases might represent the most aggressive phenotypes of a heterogeneous tumor, herein, we were interested in inter-tumor or metastases heterogeneity, using gene expression data generated by microarray technology once again. Interestingly, the predictive S3_97_-score was comparable between matched tumor and metastases, or matched metastases pairs. In only one case (Additional file 1: Table S5) classification differed between metastases and tumors.

Since data on treatment outcome are limited in the TCGA cohort originally used to develop the S3-score, we were not previously able to evaluate the effect of tyrosine kinase inhibitor (TKI) treatment on outcome prediction. Therefore, herein, we investigated the S3_97_-score using microarray data from a cohort of sunitinib-treated ccRCC patients. In this cohort, the S3_97_-score was significantly associated with progression-free survival of patients, indicating that our score enables even prediction of sunitinib outcome. Whether the same holds true for immunotherapy in the form of T cell immune checkpoint inhibitors like nivolumab needs to be investigated in future studies. Preliminary investigation of the S3_97_-score in metastatic RCC patients treated with nivolumab [22] shows that the S3_97_-score did not differ significantly in pre- and post-treatment biopsies (Additional file 1: Figure S6), indicating that there was no influence of treatment with nivolumab on the S3-score.

Taken together, we provide evidence that the S3_97_-score is more widely applicable than originally intended. To provide a more cost-effective approach for clinical application of the S3-score in individual patient samples, such as even formalin-fixed paraffin-embedded samples, we improved the S3_97_-score by reducing the number of signature genes from 97 to 15 especially for expression analyses through RT-PCR. Our improved S3_15_-score was validated using RT-PCR technology in a cohort of 108 ccRCC cases, clearly indicating that the S3_15_-score was associated with CSS in the complete cohort, as well as non-metastatic and metastatic subsets. Moreover, the S3_15_-score improves prediction of CSS by the currently clinically applied SSIGN score, which is based on clinical parameters and pathologic features. Finally, the S3_15_-score allows risk prediction in tumor and metastases tissue.

In summary, we found that our score enables valid prediction of patient outcome even if applied to different sample types (e.g., primary and metastatic tissue) and independent cohorts (e.g., patients treated with TKIs). Moreover, different platforms (RNA-seq, microarray) and technologies more appropriate for clinical utility (qRT-PCR) can be used for prediction of patient risk by the S3-score. Further prospective studies are warranted to assess the implementation of the score into clinical practice with consequences on personalized patient care.

## Conclusions

Since the stratification of patients to identify those with worse prognosis is increasingly important, especially for treatment selection, the molecular subtyping through gene expression signatures may be promising for ccRCC patients. In the present work, the clinical utility of the gene expression-based S3-score, which reflects the similarity of the tumor to its cell of origin in the nephron, was assessed in independent cohorts. The 97 gene-based S3_97_-score and a simplified 15-gene RT-PCR-based S3_15_-score are significantly associated with CSS or progression-free survival in non-metastatic and metastatic ccRCC patients, as well as in TKI-treated patients. As a result, this score, as a promising, cost-effective, and robust diagnostic tool, enables the risk stratification of patients with ccRCC in clinical practice in the non-metastatic, metastatic, and sunitinib-treated setting.

## Additional file

Additional file 1: Supplementary data including supplementary methods, tables and figures. (PDF 1124 kb)
