# Supplementary material for: Clinical utility of the S3-score for molecular prediction of outcome in non-metastatic and metastatic clear cell renal cell carcinoma
Source: BMC Med. 2018 Jul 5;16:108. doi: 10.1186/s12916-018-1088-5 (PMC6033218; doi:10.1186/s12916-018-1088-5)
Supplement: Supplementary file 1 — Supplementary data including supplementary methods, tables and figures. (PDF 1124 kb) [file 12916_2018_1088_MOESM1_ESM.pdf]

Supplementary Data (Büttner et al., Clinical utility of the S3-score for molecular prediction of outcome in non-metastatic and metastatic clear cell renal cell carcinoma)

Supplementary Methods

Supplementary Tables S1-S5

Supplementary Figures S1-S6

Supplementary Methods

Study cohorts

Primary tumor samples with clear cell RCC histology (n=108), as well as metastases samples (n=22), of patients treated at the Department of Urology, University Hospital Tuebingen, Germany, were collected. Patients' characteristics, clinicopathological features, and survival data are given in Table 1. Further details about metastases are summarized in Supplementary Table S1. Use of the tissue was approved by the ethics committee of the University of Tuebingen and informed written consent was provided by each subject prior to surgical resection. Surgically resected ccRCC tissues were classified according to the seventh edition of the Union Internationale Contre le Cancer/American Joint Committee on Cancer system (2009). Survival end-point was cancer-specific survival (CSS) defined as the time from surgery to death or to last date of follow-up if alive. Data for patients who died from other causes than ccRCC disease were considered censored at the time of death. None of the patients received any kind of neoadjuvant therapy before surgery, neither immune- nor chemotherapy.

Additionally, publicly available gene expression data from The Cancer Genome Atlas (TCGA) [1] from a cohort of ccRCC patients (n=463, as described in [2]), were used as development cohort for re-designing the S3-score calculation model. Kaplan-Meier curves of cancer-specific survival for each cohort are shown in (Supplementary Fig.S2). Moreover, publicly available gene expression data from an independent cohort of sunitinib treated ccRCC patients (n=53) [3] were used for evaluation in the present study. Supplementary Fig.S1 shows an overview about the workflow of data analyses including the different cohorts and technologies used in the present study.

Gene expression analyses

High quality total RNA was isolated from fresh-frozen ccRCC and metastasis tissue using the mirVana™ miRNA Isolation Kit (Life Technologies) as previously described [4, 5]. Genome-wide transcriptome analyses were performed using the Human Transcriptome Array HTA 2.0 (Affymetrix) according to the manufacturer's protocol. Further processing of microarray data were performed as previously described [5]. Array quality control as well as preprocessing of microarray data using Robust Multi-array Average (RMA) were conducted by Affymetrix Expression Console (Build 1.4.1.46). RMA was applied separately to each of the two sample types (ccRCC (n=52) and metastases (n=22)). The accession number for data of metastases and primary ccRCC at the European Genome-phenome Archive (EGA) (www.ebi.ac.uk/ega/home), which is hosted by the EBI and the CRG, is EGAS00001001176. Gene expression data (generated using the HuGene 1.0ST Affymetrix array) from 53 sunitinib treated ccRCC patients [3] were downloaded from ArrayExpress (E-MTAB-3267). The set of arrays was preprocessed using the Robust Multiarray Average implementation from the R-package oligo.

Quantitative real-time PCR (RT-PCR) was performed using TaqMan technology on a BioMARK System (Fluidigm) as described previously [4, 5]. TaqMan gene expression assays for 97 genes of the S3-score, as well as 5 genes used for normalization were purchased from Life Technologies (further details about the assays purchased from Life Technologies are available upon request).

Statistical analyses

Statistical tools

All statistical analyses were performed with R-3.3.3 including additional packages beeswarm_0.2.3, glmnet_2.0-5, partykit_1.1-1, oligo_1.38.0, pamr_1.55, and survival_2.41-2 [6–14]. All statistical tests were two-sided. Statistical significance was defined as $P < 0.05$.

Selection of normalization genes for RT-PCR

Candidate genes for RT-PCR normalization were identified based on RNA-Seq data from the development cohort (463 samples, see [2]) and HTA 2.0 microarray measurements in 37 ccRCC samples from the ccRCC cohort 1. Only isoforms (RNA-seq) or genes (HTA 2.0) with mean expression above the mean of all isoforms/genes were considered. Further, optimal assays should be available at Life Technologies. We applied the NormFinder software [15] to rank isoforms/genes according to expression stability across samples. Five assays were selected for normalization with Hs00191307_m1 (CIAO1), Hs00171309_m1 (ELAVL1), Hs00199190_m1 (SF3B2) selected based on RNA-Seq and Hs00999748_m1 (PRDM10), Hs00205849_m1 (BRD1) selected based on microarray data. Assays representing the 97 signature genes were divided randomly on three plates, each assay was measured in duplicate. Duplicate measurements were averaged and then normalized by subtracting the median of the normalization genes.

Calculation of the S3-score based on interprofile correlations

Interprofile correlation-based S3-scores were calculated as previously described [2]. Briefly, Spearman's correlation coefficients were computed between each tumor and each of the eight nephron regions by means of the 97 signature genes. Subsequently, the eight correlation coefficients were centered and standardized per tumor (z-scores). The z-score assigned to the S3 region constituted the S3-score of a tumor. The correlation-based calculation of the $S3_{97}$-score was used for samples for which genome-wide transcriptome expression measurements generated by RNA-Seq or microarray were available.

Development of a S3-score calculation model for use of RT-PCR data

For use of RT-PCR measurements a new calculation model of the S3-score was developed. A linear model was created that reconstructs the correlation-based S3-scores in the development cohort [2] by means of expression values of the genes from the signature set. Prior to model selection the set of predictor variables was filtered. To ensure minimum failure rates in future applications, all assays that failed (i.e. both measurements in a sample failed) minimum once were excluded. Moreover, we used only genes that were comparable with respect to mean expression and variation of expression between the development cohort (RNA-Seq) and the extended validation cohort (RT-PCR). To be precise, for each sample gene expression values were normalized by subtracting the median of the five normalization genes yielding Δlog_2RPKM (development cohort) and ΔCt values (extended validation cohort), see Supplementary Fig.S3. Genes with absolute difference in mean below 1.0 and absolute difference in standard deviation below 0.5 were kept. The resulting set of variables used for model selection included 41 genes.

The glmnet R package was applied to fit a linear model regularized by lasso penalty on the 41 genes to reconstruct the S3-scores in the development cohort. Model selection was conducted on normalized RPKM values (Δlog_2RPKM). The regularization parameter lambda was determined from averaged error curves that were obtained from repeated cross validations (500 times). Except standardization of response and predictor variables, the functions glmnet and cv.glmnet were run with default parameter settings. As suggested by glmnet, the simplest model with a cross validation error within one standard deviation of the minimum cross validation error was selected (Supplementary Table S3). This final model included 15 genes. Scores produced by this model are referred to as $S3_{15}$-scores.

In our previous work [2] we had defined a cut-off (-0.167) to distinguish between cases of high and low risk based on the S3-score. This cut-off is intended for $S3_{97}$-scores that were calculated using the correlation-based approach and hence has been used in this work to dichotomize the cohorts that were measured with microarrays. For cohorts measured with

RT-PCR we determined a separate cut-off. Using the conditional inference tree framework with endpoint CSS from the R-package partykit samples were partitioned based on the $S3_{15}$-score. The resulting cut-off (-0.249) differed only slightly from the original cut-off. From the 108 samples of the validation cohort two samples would have been grouped differently if the original cut-off had been used.

ClearCode34

The ClearCode34 classifier as introduced by Brooks et al. [16] was applied on the matched tumor and metastases samples for which genome-wide expression data measured by HTA 2.0 microarrays were available. A trainings data set for classification by the prediction analysis of microarrays (PAM) method was kindly provided by the authors (pers. Comm., S.A. Brooks, Chapel Hill, NC, USA). Gene GALNT4 was not included on the HTA 2.0 microarray and therefore disregarded. Before using PAM, gene expression values were median centered across samples.

Survival analyses

Cancer-specific survival (CSS) was used as an endpoint in survival analyses involving the development cohort (ccRCC TCGA, see [2]) as well as our ccRCC cohorts 1 and 2. CSS was defined as the time from surgery to death or last date of follow-up if alive. Data for patients who died from other causes than ccRCC disease were considered censored at the time of death. Progression-free survival was used as endpoint in the survival analysis of the cohort of sunitinib treated ccRCC patients, for details see [3]. Survival analyses for both endpoints were conducted by Kaplan-Meier curves and corresponding log-rank tests. Comparisons of Cox models were performed by analysis of deviance.

SSIGN-score

The stage, size, grade, necrosis (SSIGN) score was calculated as denoted in Zigeuner et al. [17]. Scores of 0-1 as well as scores of 10 or greater were pooled together, respectively. One sample was excluded from survival analyses involving SSIGN-score as no metastasis status information was available. Further, two tumors with assigned tumor grade "2-3" were considered as grade "3" tumors in SSIGN-score calculation.

Supplementary Table S1: Patient demographics and metastatic sites of metastases derived from ccRCC (n=22 with available microarray data; n=16 with available RT-PCR data).

		Metastases Set 1 (n=22 from 15 patients§)		Metastases Set 2 (n=16 from 14 patients§)	
		n, value	%	n, value	%
**Sex**	**Male**	12	80.00%	11	78.57%
	**Female**	3	20.00%	3	21.43%
**Age (year) at metastasis resection**	**Median (range)**	64 (50 – 77)		66 (51-77)	
**Metastatic site**	**lymph node***	10	52.63%	7	43.75%
	**adrenal gland**	3	15.79%	2	12.50%
	**local recurrence**	2	10.53%	2	12.50%
	**abdominal wall/cutaneous**	2	10.53%	1	6.25%
	**ileum**	1	5.26%	1	6.25%
	**liver**	1	5.26%	1	6.25%
	**shoulder**	1	5.26%	1	6.25%
	**thorax**	1	5.26%	1	6.25%
	**omentum**	1	5.26%	0	0.00%

§ 5 patients from our ccRCC cohort 1 and from our extended ccRCC cohort 2 (Table 1) with matched primary tumor and metastases samples were included and indicated as P1-P5 in Figure 3A and B. All other metastases are derived from independent patients that are not part of our ccRCC cohorts listed in Table 1.

* including 4 lymphnode regions of one patient

Supplementary Table S2: Univariate Cox regression for cancer-specific survival in the ccRCC cohort 1 (n=52), as well as analyses of progression-free survival in patients after treatment with sunitinib (n=53).

**Univariate analyses**	**variable**	**level**	**no. of cases**	**HR (95%CI)**	**P-value (Log-rank test)**	**c-index**
**ccRCC cohort 1**	**$S3_{97}$-score**	high	36	1 (Ref.)	3.29E-02	0.65
		low	16	2.89 (1.04 – 8.04)		
**Sunitinib treated ccRCC patients**	**$S3_{97}$-score**	high	33	1 (Ref.)	2.17E-02	0.60
		low	20	2.14 (1.10 – 4.16)		

Abbreviations: CI, confidence interval; HR, Hazard Ratio; c-index, Harrell`s c-index; Ref., Reference level. $S3_{97}$-scores were determined based on gene expression data measured by microarrays.

Supplementary Table S3: Genes included in the linear model for calculation of the $S3_{15}$-score and corresponding regression coefficients.

variable	coefficient
(Intercept)	-0.011639907
FTCD	0.02099844
NAPSA	0.01695184
PDZK1IP1	0.050775946
DPEP1	0.008775838
DEFB1	-0.026493293
ARHGDIB	-0.027795375
MIOX	0.079704719
CTGF	-0.069596024
ANPEP	0.077325565
CRYAB	0.008904111
MGP	-0.034015862
TMEM27	0.065900548
MAL	-0.001652004
EPS8L1	-0.023210981
SLPI	-0.044062178

Supplementary Table S4: Association of patient characteristics and clinicopathological parameters with "high" and "low" groups, as identified by the $S3_{15}$-score in the extended ccRCC cohort 2. Distributions are shown in Supplementary Fig. S5.

parameter	categories[†]	P-value (Fisher's exact test)
Primary tumor	T1/ T2/ T3	1.17E-01
Regional lymph node	N0/ N1/ N2	4.02E-02
Distant metastasis	M0/ M1	7.18E-02
Fuhrman grade	G1/ G2/ G3	6.31E-05
Tumor necrosis	yes/ no	9.45E-02
Gender	female/ male	6.24E-01

[†]Tumors with grade "G2-3" and "G4" were added to "G3". Tumors with no grading information or metastasis status "MX" were disregarded.

Supplementary Table S5: Concordance of S3-scores in matched tumor and metastases or metastases derived from the same patient, as well as regions from one metastasis. Results are compared to risk prediction using the ClearCode34 signature.

Patient*	Metastatic site	$S3_{97}$-score		ClearCode34	
		tumor	metastasis	tumor	metastasis
P1	lymph node	high	high	ccA	ccA
	adrenal gland		high		ccA
P2	lymph node	high	low	ccA	ccB
P3	lymph node	high	high	ccA	ccA
P4	lymph node	low	low	ccB	ccB
P5	adrenal gland	high	high	ccA	ccA
P6†	lymph node		low		ccB
	lymph node		low		ccB
	lymph node		low		ccB
	lymph node		low		ccB
P7	local recurrence		high		ccA
	local recurrence		low		ccA
P8	omentum		low		ccB
	abdominal wall/cutaneous		low		ccB
	abdominal wall/cutaneous		low		ccA

† For patient P6, four regions of one metastasis were analyzed.

***** Patients P1-P5 are metastatic patients included in our ccRCC cohort 1 and in our extended ccRCC cohort 2

Supplementary Figure S1

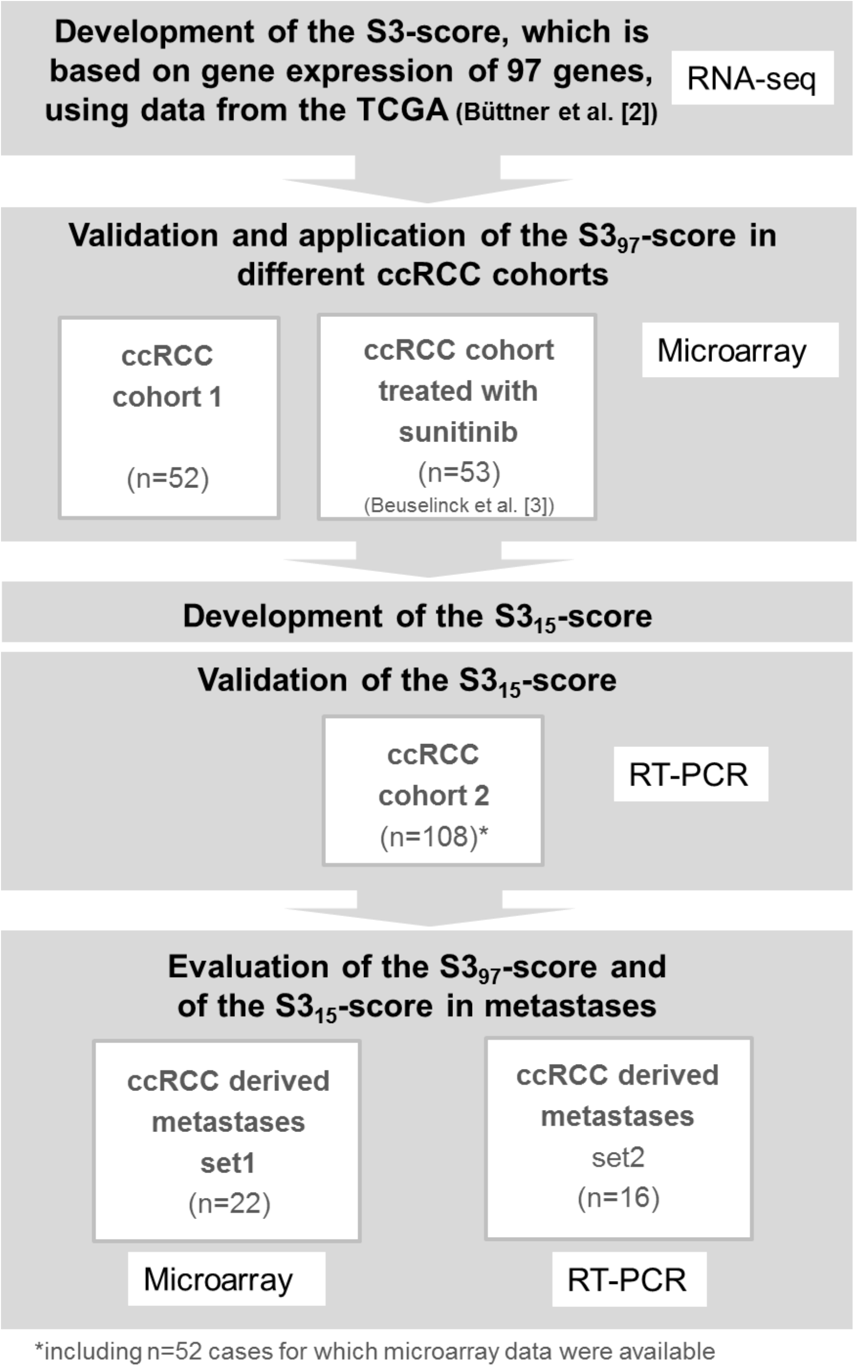

Overview about the workflow of data analyses including different cohorts and technologies (microarray and RT-PCR) used in the present study.

Supplementary Figure S2

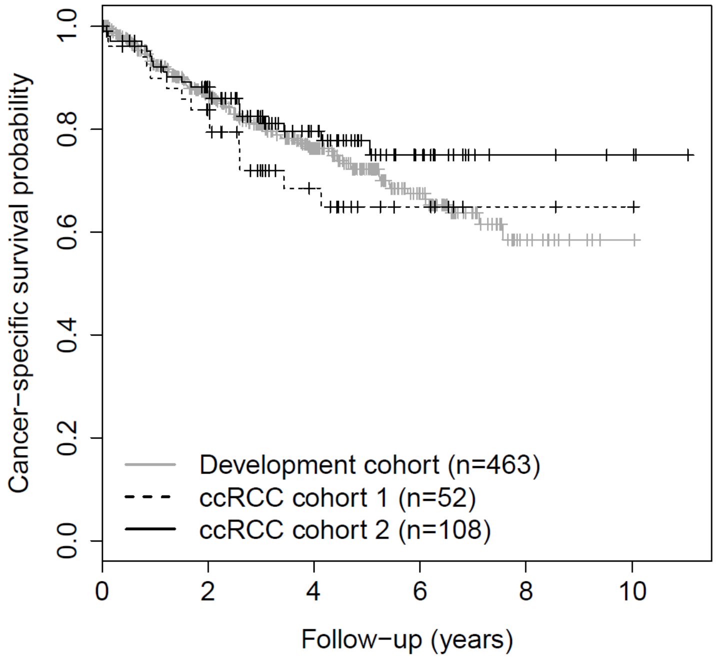

Kaplan-Meier curves of cancer-specific survival for the TCGA and ccRCC cohorts 1 and 2 are shown.

Supplementary Figure S3

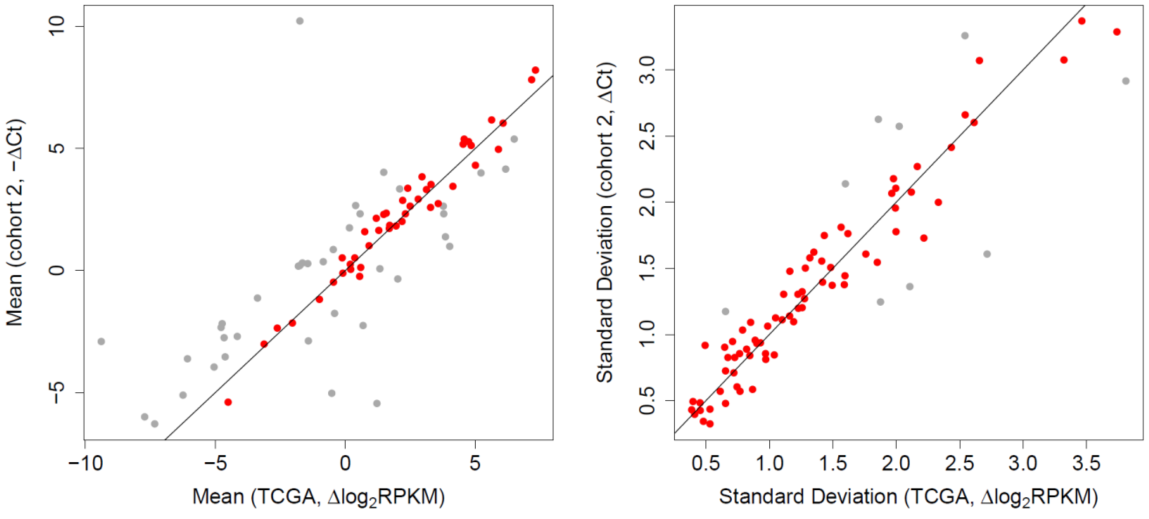

Correlation between mean gene expression (left panel) and variation of expression (right panel) between the TCGA cohort and our validation cohort. For model development only genes (marked in red) were considered that were comparable with respect to mean expression (absolute difference < 1.0) and variation of expression (absolute difference in standard deviation < 0.5) between the RNA-seq data in the TCGA cohort (which was used to develop the S3-score) and the RT-PCR values. Both data sets were normalized by subtracting the median of the five normalization genes per sample.

Supplementary Figure S4

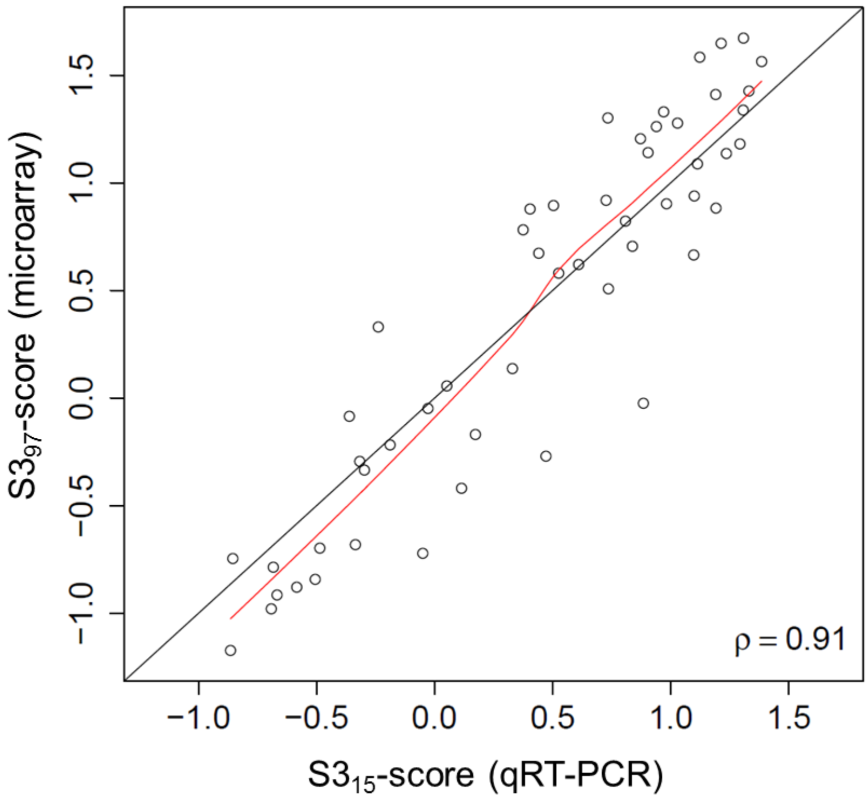

Scatterplot of the S3-scores determined through microarray or RT-PCR in ccRCC cohort 1 (n=52) along with Spearman’s rank correlation coefficient. The relationship between both scores is highlighted by the red trend line obtained by local polynomial regression fitting.

Supplementary Figure S5

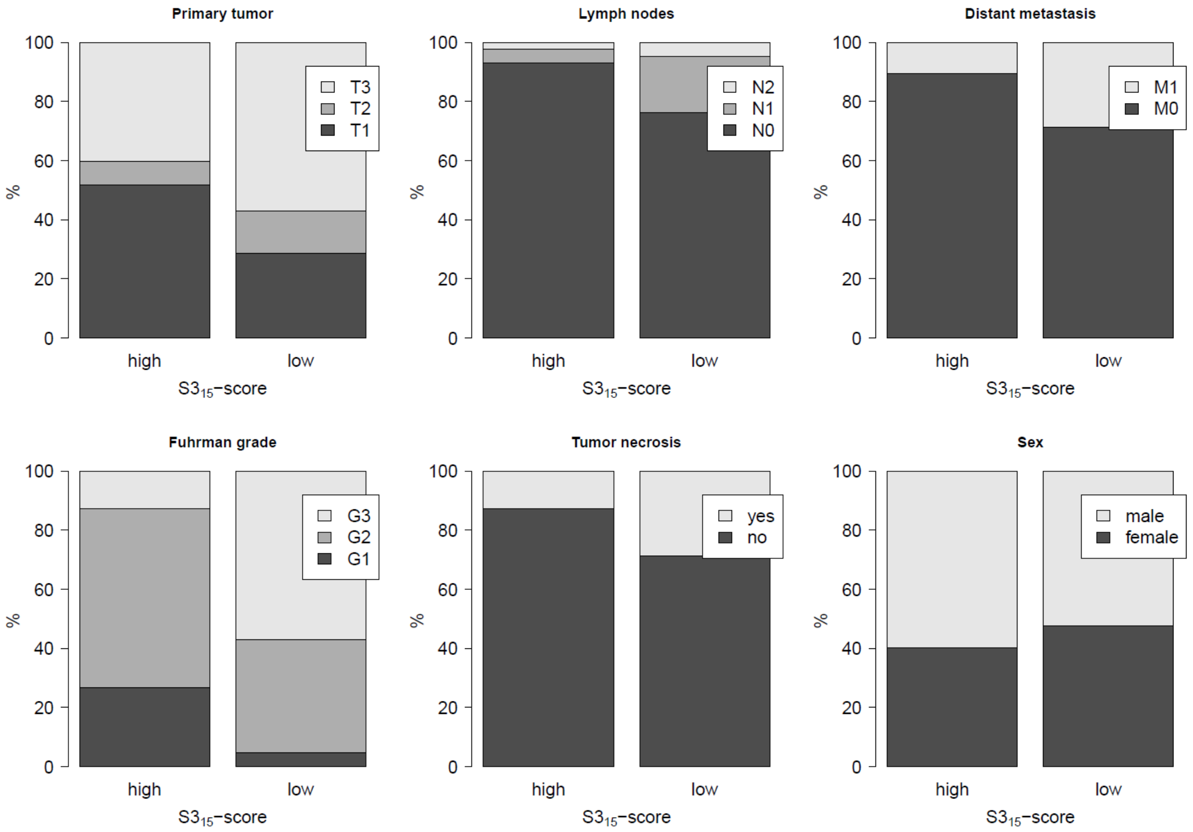

Distribution of patient characteristics and clinicopathological parameters in the “high“ and “low“ groups, as identified by the S3$_{15}$-score in the extended ccRCC cohort 2. Associated P-values are shown in Table S4. Tumors with grade “G2-3” and “G4” were added to “G3”. Tumors with no grading information or metastasis status “MX” were disregarded.

Supplementary Figure S6

A

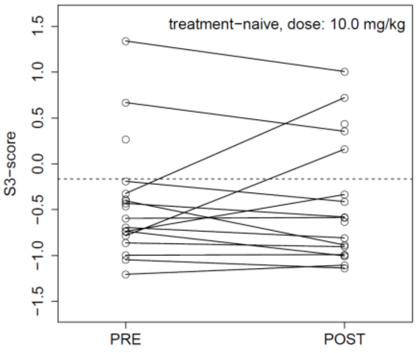

B

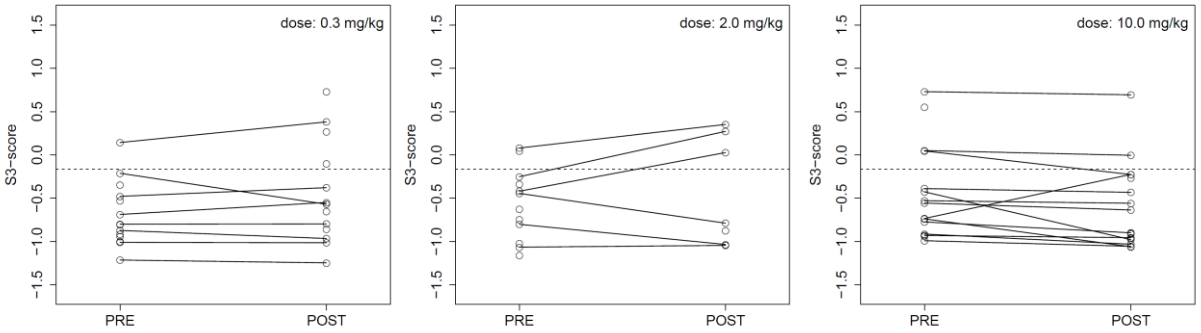

Evaluation of the S3-score in metastatic RCC patients treated with nivolumab. The S3-score was calculated based on the 97 signature genes in a cohort of patients that were treated with nivolumab intravenously every 3 weeks at 10 mg/kg to treatment-naïve patients (A), and at 0.3, 2, or 10 mg/kg to previously treated (B) patients with mRCC. The $S3_{97}$-score did not differ significantly in pre- and post-treatment biopsies irrespective whether patients were treatment-naïve (A) or not (B). Gene expression data (generated using the HG-U219 array plate, Affymetrix) from mRCC patients [18] were downloaded from ArrayExpress (E-MTAB-3218) and were pre-processed using the Robust Multiarray Average (RMA) implementation from the R-package oligo.

References

1. Cancer Genome Atlas Research Network. Comprehensive molecular characterization of clear cell renal cell carcinoma. Nature. 2013;499:43–9. doi:10.1038/nature12222.
2. Büttner F, Winter S, Rausch S, Reustle A, Kruck S, Junker K, et al. Survival Prediction of Clear Cell Renal Cell Carcinoma Based on Gene Expression Similarity to the Proximal Tubule of the Nephron. Eur Urol. 2015;68:1016-1020. doi:10.1016/j.eururo.2015.05.045.
3. Beuselinck B, Job S, Becht E, Karadimou A, Verkarre V, Couchy G, et al. Molecular subtypes of clear cell renal cell carcinoma are associated with sunitinib response in the metastatic setting. Clin Cancer Res. 2015;21:1329–39. doi:10.1158/1078-0432.CCR-14-1128.
4. Fisel P, Kruck S, Winter S, Bedke J, Hennenlotter J, Nies AT, et al. DNA methylation of the SLC16A3 promoter regulates expression of the human lactate transporter MCT4 in renal cancer with consequences for clinical outcome. Clin Cancer Res. 2013;19:5170–81. doi:10.1158/1078-0432.CCR-13-1180.
5. Winter S, Fisel P, Büttner F, Rausch S, D'Amico D, Hennenlotter J, et al. Methylomes of renal cell lines and tumors or metastases differ significantly with impact on pharmacogenes. Sci Rep. 2016;6:29930. doi:10.1038/srep29930.
6. Eklund A. beeswarm: The Bee Swarm Plot, an Alternative to Stripchart. R package version 0.2.3. 2016:https://CRAN.R-project.org/package=beeswarm.
7. Friedman J, Hastie T, Tibshirani R. Regularization Paths for Generalized Linear Models via Coordinate Descent. J. Stat. Soft. 2010. doi:10.18637/jss.v033.i01.
8. Hothorn T, Hornik K, Zeileis A. Unbiased Recursive Partitioning: A Conditional Inference Framework. Journal of Computational and Graphical Statistics. 2006;15:651–74.
9. Hothorn T, Zeileis A. partykit: A Modular Toolkit for Recursive Partytioning in R. Journal of Machine Learning Research. 2015;16:3905–9.
10. Hastie T, Tibshirani R, Narasimhan B, Chu G. pamr: Pam: prediction analysis for microarrays. R package version 1.55. 2014:https://CRAN.R-project.org/package=pamr.
11. Therneau T. *A Package for Survival Analysis in S*. version 2.38. 2015:https://CRAN.R-project.org/package=survival.
12. Therneau TM, Grambsch PM. *Modeling Survival Data: Extending the Cox Model*: Springer, New York; 2000.
13. Carvalho BS, Irizarry RA. A framework for oligonucleotide microarray preprocessing. Bioinformatics. 2010;26:2363–7. doi:10.1093/bioinformatics/btq431.
14. R Core Team. R: A language and environment for statistical computing. R Foundation for Statistical Computing, Vienna, Austria. 2014.
15. Andersen CL, Jensen JL, Orntoft TF. Normalization of real-time quantitative reverse transcription-PCR data: a model-based variance estimation approach to identify genes suited for normalization, applied to bladder and colon cancer data sets. Cancer Res. 2004;64:5245–50. doi:10.1158/0008-5472.CAN-04-0496.
16. Brooks SA, Brannon AR, Parker JS, Fisher JC, Sen O, Kattan MW, et al. ClearCode34: A prognostic risk predictor for localized clear cell renal cell carcinoma. Eur Urol. 2014;66:77–84. doi:10.1016/j.eururo.2014.02.035.
17. Zigeuner R, Hutterer G, Chromecki T, Imamovic A, Kampel-Kettner K, Rehak P, et al. External validation of the Mayo Clinic stage, size, grade, and necrosis (SSIGN) score for clear-cell renal cell carcinoma in a single European centre applying routine pathology. Eur Urol. 2010;57:102–9. doi:10.1016/j.eururo.2008.11.033.
18. Choueiri TK, Fishman MN, Escudier B, McDermott DF, Drake CG, Kluger H, et al. Immunomodulatory Activity of Nivolumab in Metastatic Renal Cell Carcinoma. Clin Cancer Res. 2016;22:5461–71. doi:10.1158/1078-0432.CCR-15-2839.
